# ED visits, hospital admissions and treatment breaks in head/neck cancer patients undergoing radiotherapy

**DOI:** 10.3389/fonc.2023.1147474

**Published:** 2023-03-01

**Authors:** Shareen Patel, Benjamin J. Rich, Leif-Erik D. Schumacher, Zoukaa B. Sargi, Melissa Masforroll, Cyrus Washington, Deukwoo Kwon, Maria A. Rueda-Lara, Laura M. Freedman, Stuart E. Samuels, Matthew C. Abramowitz, Michael A. Samuels, Ruben Carmona, Gregory A. Azzam

**Affiliations:** ^1^ Department of Radiation Oncology, Sylvester Comprehensive Cancer Center, University of Miami Miller School of Medicine, Miami, FL, United States; ^2^ Department of Otolaryngology, University of Miami Miller School of Medicine, Miami, FL, United States; ^3^ Department of Medicine, Florida International University, Miami, FL, United States; ^4^ Department of Public Health Sciences, Biostatistics and Bioinformatics Shared Resource, Sylvester Comprehensive Cancer Center, University of Miami Miller School of Medicine, Miami, FL, United States; ^5^ Department of Psychiatry, University of Miami Miller School of Medicine, Miami, FL, United States

**Keywords:** head/neck cancer, radiation therapy, hospital admissions, ED visits, treatment breaks, head and neck neoplasms, radiotherapy, hospital admissions

## Abstract

**Objectives:**

Radiation therapy (RT) is an integral part of treatment of head/neck cancer (HNC) but is associated with many toxicities. We sought to evaluate sociodemographic, pathologic, and clinical factors associated with emergency department (ED) visits, hospital admissions (HA), and RT breaks in HNC patients undergoing curative-intent RT.

**Methods:**

We completed a Level 3 (Oxford criteria for evidence-based medicine) analysis of a cohort of HNC patients who underwent curative-intent RT at our institution from 2013 to 2017. We collected demographic characteristics and retrospectively assessed for heavy opioid use, ED visits or HA during RT as well as RT breaks. Treatment breaks were defined as total days to RT fractions ratio ≥1.6. Multivariable stepwise logistic regression analyses were done to determine the association of various sociodemographic, pathologic, and clinical characteristics with ED visits, HA and RT treatment breaks.

**Results:**

The cohort included 376 HNC patients (294 male, 82 female, median age 61). On multivariable analysis, significant factors associated with ED visits during RT were heavy opioid use and black race. Receipt of concomitant chemotherapy was the only factor associated with hospital admissions during RT. Advanced age, lower socioeconomic class, glandular site, and receipt of chemotherapy were all independently associated with RT breaks. Lower cancer stage and lack of substance abuse history were independently associated with lack of treatment breaks.

**Conclusion:**

HNC patients with factors such as heavy opioid use, Black race, receipt of concomitant chemotherapy, and lower socioeconomic class may require closer monitoring during RT.

## Introduction

Head and neck cancer accounts for approximately 900,000 cases and over 400,000 deaths annually (GLOBOCAN 2020 data) (1). Radiation therapy (RT) is an important part of the multidisciplinary management of head and neck cancer. Unplanned hospitalizations and emergency department (ED) visits during the radiation treatment course can lead to treatment breaks, disproportionately affecting certain vulnerable populations and leading to a financial burden on patients and the healthcare system (2). Overall survival and cancer-specific survival is significantly decreased for head and neck cancer patients hospitalized during radiation therapy, with dehydration and fever the leading causes of admission (3). Moreover, adherence to the radiation treatment timeline is important as unplanned RT breaks and prolongation of the RT period is associated with worse survival and locoregional control of disease, possibly due to rapid repopulation (4–6).

Quality of life scores significantly decrease as patients experience oral complications during or after RT, with pain a major contributor (7). Pain can be prevalent throughout the radiation treatment course and, in some patients, persist 6-12 months post-RT (8). Painful sequelae such as oral mucositis during curative-intent RT for head and neck cancer is a common reason for hospitalizations during treatment (9). In order to optimize pain control amongst head and neck cancer patients, opioids are commonly prescribed (10, 11). Head and neck cancer patients treated with radiotherapy are at risk for long-term opioid use (12, 13), but predicting long-term opioid use is difficult (11).

Although pain and resulting opioid use play a large role in the treatment course of head and neck patients, prior studies evaluating risks for ED visits, unplanned hospitalizations, and treatment breaks in this population have failed to take these important components into account (11). In this study, we sought to analyze the occurrence of ED visits, unplanned hospitalizations, and radiation treatment breaks in head and neck cancer patients undergoing curative-intent radiation therapy in relation to pain and opioid use as well as other clinical, treatment and socioeconomic characteristics.

## Materials and methods

This single-institution study was approved by the Institutional Review Board (IRB). We retrospectively identified a group of patients with head and neck cancer at our institution from the Tumor Registry who were treated with curative-intent external beam radiotherapy from 2013 to 2017. Additional inclusion criteria for the cohort included: (a) received RT at our institution, (b) did not have persistent disease or recurrent disease within 18 months, (c) had no history of chronic opioid use for non-cancer pain before cancer presentation or diagnosis, (d) received RT to the primary disease site and (e) had non-metastatic disease. Patients were excluded if they had received prior irradiation or other treatment not part of the current treatment course (indicating recurrent disease).

The electronic medical record (EMR) of eligible patients was reviewed and clinical data was collected in a Research Data Electronic Capture (REDCap) database. Sociodemographic, pathologic, and clinical factors were collected for each patient (**Table 1**). Additionally, we recorded opioid use, hospitalizations and ED visits within the radiation treatment period and total days to complete radiation therapy from the EMR. Each patient’s chart was reviewed for hospitalizations and ED visits in our hospital system as well as documentation of hospitalizations or ED visits outside of our hospital system. Documentation reviewed included weekly on-treatment notes during RT. Planned hospitalizations solely for chemotherapy infusion were excluded unless the hospitalization was extended beyond two days for supportive care related to RT side effects. ED visits leading to a hospital admission were counted as a hospitalization, but not an ED visit. We calculated the ratio of total days from start to completion of RT divided by the fractions completed to assess for a prolonged RT course. Substantial treatment breaks were defined as total days to RT fractions ratio ≥1.6. The ratio cutoff of ≥1.6 days/fraction was chosen based on work by Ho et al. demonstrating a survival significance at this days/fraction ratio (14). In certain cases where an opioid was prescribed, but the patient reported not using opioids, no opioid dose was recorded. Heavy opioid use was defined as >30 morphine milligram equivalents (MME) daily as used elsewhere in the literature (15, 16).

**Table 1:** Patient characteristics.

Characteristic		Patients (N=376)
Age in years – median		61
Sex – n (%)		
	Female	82 (21.8)
	Male	294 (78.2)
Race/Ethnicity – n (%)		
	Non-Hispanic White	208 (55.3)
	Hispanic White	117 (31.1)
	Black	27 (7.2)
	Asian	5 (1.3)
	Native American	2 (0.5)
	Other/Unknown	17 (4.5)
Income class – n (%)		
	Lower	119 (31.6)
	Middle	196 (52.1)
	Upper	51 (13.6)
	Unknown/International Patient	10 (2.7)
Primary Language – n (%)		
	English	284 (75.5)
	Spanish	84 (22.3)
	Other	8 (2.1)
Marital Status – n (%)		
	Married	258 (68.6)
	Not Married	113 (30.1)
	Unknown	5 (1.3)
Living Situation – n (%)		
	Alone	37 (9.8)
	With Family	248 (66.0)
	Unknown	91 (24.2)
Dependent Children – n (%)		
	Yes	116 (30.9)
	No	151 (40.2)
	Unknown	109 (29.0)
Employment – n (%)		
	Employed	217 (57.7)
	Unemployed	107 (28.5)
	Unknown	52 (13.8)
Insurance – n (%)		
	Private	188 (50.0)
	Medicare	157 (41.8
	Medicaid	23 (6.1)
	None Documented	8 (2.1)
Smoking or Tobacco History – n (%)		
	Never	171 (45.5)
	Former	184 (48.9)
	Current	20 (5.3)
Substance Abuse History – n (%)		
	Never	324 (86.2)
	Former	26 (6.9)
	Current	26 (6.9)
History of Psychiatric Disorder – n (%)		
	No	344 (91.5)
	Yes	32 (8.5)
History of chronic pain condition – n (%)		
	No	254 (67.6)
	Yes	122 (32.4)
Cancer Location – n (%)		
	Hypopharynx	7 (1.9)
	Larynx	66 (17.6)
	Nasal Cavity	21 (5.6)
	Nasopharynx	13 (3.5)
	Oral Cavity	43 (11.4)
	Oropharynx	190 (50.5)
	Parotid or Submandibular Gland	26 (6.9)
	Ethmoid or Maxillary Sinus	10 (2.7)
AJCC 7^th^ ed. Cancer Stage – n (%)		
	I	47 (12.5)
	II	46 (12.2)
	III	78 (20.7)
	IV	191 (50.8)
Treatment Modality – n (%)		
	Radiation	376 (100.0)
	Chemotherapy	152 (40.4)
	Surgery	181 (48.1)
Total Radiation Dose – n (%)		
	≤50 Gy	13 (3.5)
	>50 to 60 Gy	95 (25.3)
	>60 to 70 Gy	266 (70.7)
	>70 Gy	2 (0.5)
Gabapentin Use – n (%)		
	No	355 (94.4)
	Yes	21 (5.6)
Mouthwash Use – n (%)		
	No	83 (22.1)
	Yes	293 (77.9)
Patients Admitted to Hospital– n (%)		
	No	320 (85.1)
	Yes	56 (14.9)
Number of Hospital admissions – n (%)		
	0	320 (85.1)
	1	41 (10.9)
	2	11 (2.9)
	3+	4 (1.1)
Total days of hospital admission(s) – n (%)		
	1	4 (1.1)
	2	9 (2.4)
	3	9 (2.4)
	4+	34 (9.0)
Patients with ED visit – n (%)		
	No	356 (94.7)
	Yes	20 (5.3)
Median Days/RT Fractions Ratio		1.38
Days/RT Fractions Ratio		
	<1.6	357 (94.9)
	≥1.6	19 (5.1)

AJCC, American Joint Committee on Cancer; ED, Emergency Department; RT, Radiation Therapy.

## Statistical methods

Continuous variables were summarized with descriptive statistics and categorical variables were summarized using counts and proportions. Univariable and multivariable logistic regression analyses were performed to determine the association of socioeconomic class, race, ethnicity, age, marital status, gender, primary language, employment, living situation, chemotherapy, opioid use, non-opioid substance use, history of substance use, chronic pain condition, psychiatric disease, cancer site or cancer stage with ED visits, hospital admissions and RT treatment breaks. Odds ratios (OR) were collected and a p-value <0.05 was considered statistically significant. For the multivariable analysis, we used stepwise variable selection. Statistical analyses were performed with the statistical software package R, version 4.0.5 (R foundation for Statistical Computing).

## Results

The institution tumor registry contained a total of 678 patients with head and neck cancer treated with external beam radiation therapy from 2013 to 2017. Our cohort included 376 patients after excluding patients who did not meet our inclusion and exclusion criteria. **Table 1** contains patient characteristics of the cohort. The cohort consisted of 78.2% males and 21.8% females with a median age of 61 years. The patients included non-Hispanic white (55.3%), Hispanic white (31.1%), Black (7.2%), Asian (1.3%), Native American (0.5%), and other (4.5%). The majority of patients were married (68.6%), middle class (52.1%), English-speaking (75.5%), living with family members (66%), employed (57.7%), without history of substance abuse (86.2%) or history of psychiatric disorder (91.5%), and with locally advanced disease (71.5% stage III-IV). The most common primary cancer sites were oropharynx (50.5%) and larynx (17.6%). The median radiation therapy dose received was 66 Gy. Of the cohort, 40.4% of patients received chemotherapy during the treatment course and 48.1% of patients received radiation therapy pre- or post-operatively.

In the cohort, 14.9% of the patients had at least one unplanned hospital admission and 5.3% had at least one ED visit not leading to hospitalization. Of the patients who had at least one unplanned hospitalization, 41 (73.2%) patients had one admission, 11 (19.6%) patients had two admissions and 4 (7.1%) patients had three or more admissions. The majority of patients with unplanned hospital admissions had a length of stay greater than 3 days (60.7%). The reasons for unplanned hospital admissions included dehydration (51.8% of admissions), mucositis (17.8%), fever with or without neutropenia (17.8%), intractable nausea or vomiting (3.5%), non-opioid induced constipation (1.8%), diarrhea (1.8%), draining fistula (1.8%), chest pain (1.8%), and urinary tract infection (1.8%). For unplanned hospitalizations, 93% were due to treatment or cancer-related factors and 7% were non-cancer related. The diagnoses for ED visits included dehydration (40%), dysphagia (15%), neutropenic fever (10%), urinary retention (10%), shortness of breath (5%), irritation around PEG tube (5%), depression (5%), constipation (5%), and nephrolithiasis (5%). In total, 80% of ED visits were due to treatment or cancer-related factors and 20% were non-cancer related. The median days to RT fraction ratio was 1.38 days/fraction, with 19 (5.1%) of patients with a ratio ≥ 1.6 days/fraction.

A univariable and multivariable analysis was completed to determine factors associated with ED visits, hospital admissions, and RT treatment breaks. On multivariable analysis, the factors independently associated with ED visits during RT were heavy opioid use (OR 5.39, p<0.01) and black race (OR 6.93, p<0.01) (**Table 2**). Unplanned hospital admissions during RT were only independently associated with the receipt of concomitant chemotherapy (OR 9.73, p<0.01) (**Table 3**). Heavy opioid use was associated with hospital admissions on univariable analysis (OR 2.00, p=0.019), but this was not significant on multivariable analysis. On the multivariable analysis, older age (OR 1.08, p<0.01), lower socioeconomic class (OR 4.94, p<0.01), primary salivary tumor site (OR 5.39, p=0.04), and receipt of chemotherapy (OR 6.86, p=0.01) were all independently associated with RT breaks (**Table 4**). Lower cancer stage (OR 0.48, p<0.01) and lack of substance abuse history (OR 0.17, p<0.01) were independently associated with lack of treatment breaks. Other socioeconomic, pathologic and clinical treatment variables analyzed in this study did not disclose significant associations.

**Table 2:** Factors influencing ED visits.

		Univariable Analysis (UVA)		Multivariable Analysis (MVA)	
Variable	Category	OR (95% CI)	P-value	OR (95% CI)	P-value
Daily Opioid Use	≤ 30 MME	Reference			
	> 30 MME	4.50 (1.79, 12.27)	0.002	5.39 (2.07, 15.53)	<0.001
Age	One year increased	Reference			
		0.98 (0.94, 1.02)	0.352		
Sex	Female	Reference			
	Male	0.83 (0.31, 2.61)	0.723		
Race	White	Reference			
	Black	5.06 (1.54, 14.48)	0.004	6.93 (1.98, 22.30)	0.001
	Native American	NA	0.993		
	More than one race	6.19 (0.30, 51.03)	0.122		
Ethnicity	Hispanic	Reference			
	Non-Hispanic White	1.66 (0.63, 5.21)	0.335		
	Other/Unknown	NA	0.990		
Income class	Upper	Reference			
	Lower	1.47 (0.56, 3.65)	0.412		
Primary language	English	Reference			
	Spanish	0.90 (0.11, 6.63)	0.919		
Marital status	Not Married	Reference			
	Married	0.84 (0.34, 2.29)	0.720		
Employment	Unemployed	Reference			
	Employed	1.38 (0.55, 3.76)	0.499		
Insurance	None	Reference			
	Medicare	1.13 (0.45, 2.81)	0.787		
	Medicaid	0.79 (0.04, 4.11)	0.824		
	Not Documented	NE			
Smoking/tobacco history	Never	Reference			
	Former	0.32 (0.11, 0.87)	0.035		
	Current	0.93 (0.05, 4.89)	0.948		
Substance abuse history	Yes	Reference			
	No	0.90 (0.29, 3.97)	0.876		
Substance abuse at initial visit	Yes	Reference			
	No	1.43 (0.28, 26.31)	0.730		
Site of Disease	Oropharynx	Reference			
	Nasopharynx	3.48 (0.51, 14.28)	0.121		
	Oral Cavity	0.85 (0.13, 3.11)	0.836		
	Sinuses	NE	0.991		
	Larynx and Hypopharynx	NE	0.990		
	Parotid and Submandibular Glands	NE	0.990		
Cancer stage	One stage increased	Reference			
		1.01 (0.94, 1.06)	0.899		
Surgery	No	Reference			
	Yes	0.82 (0.33, 2.08)	0.669		
Chemotherapy	No	Reference			
	Yes	2.50 (0.90, 8.87)	0.107		
Total Radiation Dose	One unit increased	Reference			
		1.05 (0.97, 1.17)	0.294		
Pain scale	One unit increased	Reference			
		1.01 (0.92, 1.10)	0.840		

ED, Emergency Department; MME, morphine milligram equivalent; NE, not evaluatable.

**Table 3:** Factors Influencing Hospital Admissions.

		Univariable Analysis (UVA)		Multivariable Analysis (MVA)	
Variable	Category	OR (95% CI)	P-value	OR (95% CI)	P-value
Daily Opioid Use	≤ 30 MME	Reference			
	> 30 MME	2.00 (1.11, 3.57)	0.019		
Age	One year increased	Reference			
		1.00 (0.98, 1.03)	0.837		
Sex	Female	Reference			
	Male	0.81 (0.42, 1.61)	0.531		
Race	White	Reference			
	Black	0.69 (0.16, 2.08)	0.569		
	Native American	NE	0.988		
	More than one race	1.92 (0.09, 15.31)	0.575		
Ethnicity	Hispanic	Reference			
	Non-Hispanic White	0.89 (0.49, 1.61)	0.684		
	Other/Unknown	NE	0.989		
Income class	Upper	Reference			
	Lower	1.35 (0.74, 2.44)	0.309		
Primary language	English	Reference			
	Spanish	1.64 (0.86, 3.05)	0.121		
Marital status	Not Married	Reference			
	Married	0.87 (0.48, 1.62)	0.657		
Employment	Unemployed	Reference			
	Employed	1.06 (0.60, 1.91)	0.842		
Insurance	None	Reference			
	Medicare	1.45 (0.82, 2.58)	0.195		
	Medicaid	1.21 (0.34, 3.37)	0.742		
	Not Documented	NE			
Smoking/tobacco history	Never	Reference			
	Former	1.24 (0.70, 2.20)	0.453		
	Current	0.62 (0.10, 2.24)	0.531		
Substance abuse history	Yes	Reference			
	No	0.96 (0.44, 2.30)	0.915		
Substance abuse at initial visit	Yes	Reference			
	No	0.72 (0.28, 2.22)	0.521		
Site of Disease	Oropharynx	Reference			
	Nasopharynx	2.66 (0.70, 8.49)	0.114		
	Oral Cavity	0.73 (0.24, 1.78)	0.524		
	Sinuses	0.63 (0.03, 3.44)	0.662		
	Larynx and Hypopharynx	0.75 (0.32, 1.60)	0.487		
	Parotid and Submandibular Glands	0.46 (0.07, 1.60)	0.297		
Cancer stage	One stage increased	Reference			
		1.01 (0.93, 1.06)	0.720		
Surgery	No	Reference			
	Yes	0.24 (0.13, 0.43)	<0.001		
Chemotherapy	No	Reference			
	Yes	9.73 (3.86, 32.74)	<0.001	9.73 (3.86, 32.74)	<0.001
Total Radiation Dose	One unit increased	Reference			
		1.11 (1.04, 1.20)	0.003		
Pain scale	One unit increased	Reference			
		1.06 (1.00, 1.12)	0.042		

MME, morphine milligram equivalent; NE, not evaluatable.

**Table 4:** Factors influencing radiation treatment breaks.

		Univariable Analysis (UVA)		Multivariable Analysis (MVA)	
Variable	Category	OR (95% CI)	P-value	OR (95% CI)	P-value
Daily Opioid Use	≤ 30 MME	Reference			
	> 30 MME	0.78 (0.25, 2.10)	0.643		
Age	One year increased	Reference			
		1.03 (0.98, 1.08)	0.150	1.08 (1.03, 1.15)	0.006
Sex	Female	Reference			
	Male	0.77 (0.28, 2.44)	0.626		
Race	White	Reference			
	Black	1.56 (0.24, 5.88)	0.565		
	Native American	NE	0.993		
	More than one race	6.56 (0.32, 54.17)	0.111		
Ethnicity	Hispanic	Reference			
	Non-Hispanic White	0.73 (0.29, 1.93)	0.513		
	Other/Unknown	19.78 (0.76, 513.97)	0.038		
Income class	Upper	Reference			
	Lower	3.17 (1.25, 8.40)	0.016	4.94 (1.75, 15.31)	0.003
Primary language	English	Reference			
	Spanish	2.12 (0.77, 5.46)	0.127		
Marital status	Not Married	Reference			
	Married	0.39 (0.15, 0.99)	0.047		
Employment	Unemployed	Reference			
	Employed	0.41 (0.15, 1.04)	0.066		
Insurance	None	Reference			
	Medicare	2.47 (0.97, 6.78)	0.064		
	Medicaid	1.87 (0.28, 7.11)	0.424		
	Not Documented	NE			
Smoking/tobacco history	Never	Reference			
	Former	3.08 (1.15, 9.69)	0.034		
	Current	NE	0.991		
Substance abuse history	Yes	Reference			
	No	0.32 (0.12, 0.95)	0.028	0.17 (0.05, 0.59)	0.004
Substance abuse at initial visit	Yes	Reference			
	No	NE	0.990		
Site of Disease	Oropharynx	Reference			
	Nasopharynx	3.70 (0.55, 15.25)	0.105		
	Oral Cavity	0.91 (0.14, 3.32)	0.898		
	Sinuses	NE	0.991		
	Larynx and Hypopharynx	0.54 (0.08, 1.94)	0.416		
	Parotid and Submandibular Glands	2.72 (0.60, 8.94)	0.132	5.39 (0.94, 27.41)	0.044
Cancer stage	One stage increased	Reference			
		0.78 (0.53, 1.03)	0.235	0.48 (0.27, 0.83)	0.009
Surgery	No	Reference			
	Yes	0.93 (0.37, 2.45)	0.878		
Chemotherapy	No	Reference			
	Yes	1.72 (0.64, 5.43)	0.307	6.86 (1.62, 37.26)	0.015
Total Radiation Dose	One unit increased	Reference			
		1.00 (0.94, 1.08)	0.908		
Pain scale	One unit increased	Reference			
		0.88 (0.78, 0.98)	0.308		

MME, morphine milligram equivalent; NE, not evaluatable.

## Discussion

ED visits and unplanned hospitalizations during curative-intent RT for head and neck cancer can lead to significant resource utilization (9, 17)and treatment breaks, which result in worse locoregional disease control and poorer survival (5, 6). Despite the importance of minimizing unplanned hospital encounters and treatment breaks in this patient population, the literature on factors associated with these events is limited and without inclusion of pain or opioid use, both important, quantifiable factors of patient experience during RT. In this large cohort of 376 head and neck cancer patients treated with curative intent RT, heavy opioid use was independently associated with ED visits during RT (OR 5.39, p<0.001), but not unplanned hospitalizations or RT treatment breaks. Pain scores during RT were not independently associated with ED visits, unplanned hospitalizations or RT treatment breaks.

Other socioeconomic and treatment related factors were associated with these events in the cohort. Black race (OR 6.93, p= 0.001) was a significant, independent predictor of ED visits. At least one unexpected hospital admissions occurred in 14.9% of the patients in our cohort (n = 56). Unplanned hospital admissions had a significant association with receipt of concomitant chemotherapy (OR 9.73, p<0.001). 5.1% of patients in the cohort had a significant radiation treatment break. Advanced age, lower socioeconomic status, primary salivary tumor site, lower cancer stage, receipt of chemotherapy, and history of substance abuse were all independently associated with RT breaks (p<0.05).

We found a significant association between ED visits and heavy opioid use, but unplanned hospital admissions and RT breaks did not share this association. Pain was also not an independent risk factor. This may suggest patients requiring heavy opioids require extra counseling or alternative analgesics to prevent unnecessary ED visits. However, patients with heavy opioid use do not appear to be at higher risk of more serious complications leading to hospitalizations or treatment breaks.

The predictors of unplanned hospital encounters found in the present study should be taken into context with existing literature. Like the present study, chemotherapy in the treatment course of head and neck cancer patients has been previously shown to be associated with an increased number of hospital admissions (3, 18, 19). This association is not limited to head and neck patients (20). Our study is the first to report that Black race is associated with ED visits. Other studies have found treatment at a public hospital, comorbidities, radiation dose, smoking status all associated with unplanned hospitalizations (18, 21).

In our cohort, 14.9% of the patients had at least one unplanned hospital admission and 5.3% had at least one ED visit, which is consistent with existing literature showing 20%-36% of patients undergoing curative intent RT for head and neck cancer had at least one hospitalization (3) (18). Unplanned hospital encounters, in addition to treatment breaks and resulting worse cancer outcomes, lead to significant resource utilization (2, 3, 17). In the United States, a 2019 study showed hospitalizations of head and neck cancer patients had an average length of stay of 6.6 days for one admission with an average cost of $18,371 (17).

In the present study, advanced age, lower socioeconomic status, primary salivary tumor site, lower cancer stage, receipt of chemotherapy, and history of substance abuse were all independently associated with RT breaks (p<0.05). The literature reports various patient characteristics associated with treatment breaks (22–24). Age is associated with enteral feeding during RT for head and neck cancer and could explain this finding (25). Other studies have found treatment breaks are associated with lower socioeconomic status (23, 24), and this could explain the worse survival seen in head and neck patients with higher baseline financial burden undergoing RT (26). Treatment breaks could also be explained by insurance disparities described elsewhere, although this was not the case for the present study (27).

Nutritional status, hydration status, and feeding tube placement are important factors that can result in unplanned ED visits, hospital admissions and treatment breaks. Among the ED visits in this study, 60% were related to nutritional or hydration status and 52% of unplanned hospital admissions were related to nutritional or hydration status. Early PEG tube placement during head and neck radiotherapy is correlated with a reduction in weight loss and, as a result, hospitalizations for nutritional deficits (28). However, PEG tube placement comes with a variety of complications that could lead to unplanned ED visits or hospitalizations: 12% of all tubes require replacement, with infection rates of approximately 9% and significant pain in 6% of patients (29).

Our study includes several limitations in addition to biases inherent to respective studies. First, the data was collected from the EMR at a single institution. Although all available records were diligently reviewed, outside ED visits or hospitalizations may have been missed if they were not documented in the EMR at out institution. Additionally, the days of treatment to RT fractions ratio may have been affected by holidays, but given the cutoff of ≥1.6 this is unlikely to have much, if any, effect on the categorization of treatment breaks. Lastly, the cohort only included head and neck cancer patients who underwent curative-intent radiation therapy and excluded patients with persistent disease, making the data collected pertinent only to this subset of patients. This study is strengthened by a relatively large, homogenous number of patients with a robust number of variables collected.

## Conclusion

In this analysis of 376 head and neck patients receiving curative-intent RT, we found heavy opioid use to be independently associated with ED visits during RT, but not unplanned hospitalizations or RT treatment breaks. Other factors independently, significantly associated included Black race with ED visits and receipt of chemotherapy with unplanned hospital admissions during RT. RT breaks were associated with advanced age, lower socioeconomic class, primary salivary tumor site, and concomitant chemotherapy. Lower cancer stage and lack of substance abuse history were independently associated with lack of treatment breaks. Head and neck cancer patients with these factors may require extra care during the RT course to prevent ED visits, hospitalizations and treatment breaks.

## Data availability statement

The datasets presented in this article are not readily available because not available for public dissemination as per IRB. Requests to access the datasets should be directed to shareen.patel@med.miami.edu.

## Author contributions

SP, BR, GA, and MS contributed to conception and design of the study. SP, L-ES, MM organized the database. DK, RC and BR performed the statistical analysis. SP and BR wrote the first draft of the manuscript. MS and GA wrote sections of the manuscript. CW, MR-L, and ZS contributed to investigation. SS, LF, MS and MA contributed to supervision of study. All authors contributed to the article and approved the submitted version.
